# Antihypertensive Effects of Benzaldehyde in Rats: Involvement of Endothelium-Independent and Endothelium-Dependent Vasorelaxation via Prostacyclin (PGI_2_)/cAMP Pathway Activation and Calcium Channel Inhibition

**DOI:** 10.3390/ph19060945

**Published:** 2026-06-16

**Authors:** Ismail Bouadid, Adil Qabouche, Morad Hebi, Mohammed El Mesky, Jwaher Haji Alhaji, Mourad A. M. Aboul-Soud, John P. Giesy, Mohamed Eddouks

**Affiliations:** 1 Team of Ethnopharmacology and Pharmacognosy, Faculty of Sciences and Techniques, Moulay Ismail University of Meknes, BP 509, Boutalamine, Errachidia 52000, Morocco; 2 Laboratory of Drug Sciences, Faculty of Medicine, Pharmacy and Dentistry, University of Sidi Mohamed Ben Abdellah, BP 1893, Km 2.200, Road of Sidi Harazem, Fez 30070, Morocco; 3 Laboratory of Materials Engineering for the Environment and Natural Resources, Faculty of Sciences and Techniques, Moulay Ismail University of Meknes, BP 509, Boutalamine, Errachidia 52000, Morocco; 4 Department of Health Sciences, College of Applied Studies, King Saud University, P.O. Box 2455, Riyadh 11451, Saudi Arabia; 5 Center of Excellence in Biotechnology Research (CEBR), College of Applied Medical Sciences, King Saud University, P.O. Box 17 10219, Riyadh 11433, Saudi Arabia; 6 Department of Veterinary Biomedical Sciences and Toxicology Centre, Western College of Veterinary Medicine, University of Saskatchewan, Saskatoon, SK S7N 5B4, Canada; 7 Department of Integrative Biology and Center for Integrative Toxicology, Michigan State University, East Lansing, MI 48824, USA; 8 Department of Environmental Sciences, Baylor University, Waco, TX 76706, USA

**Keywords:** benzaldehyde, hypertension, vasorelaxant, PGI2, Ca^2+^ channels

## Abstract

**Aims**: Globally, cardiovascular disorders represent the foremost contributor to mortality, and elevated arterial pressure constitutes one of their principal risk determinants. Despite the availability of various treatments, optimal blood pressure control is rarely achieved. The present work was undertaken to assess the antihypertensive and vasorelaxant properties of benzaldehyde, a naturally occurring aromatic aldehyde whose role in the regulation of arterial pressure has, to our knowledge, not yet been documented. **Materials and Methods**: Antihypertensive activity was evaluated in normotensive Wistar rats and in animals rendered hypertensive by chronic administration of Nω-nitro-L-arginine methyl ester (L-NAME). Benzaldehyde was administered orally at doses of 20 and 40 mg/kg under both acute (6 h) and subacute (7-day) treatment protocols. Arterial pressure was recorded non-invasively by means of a tail-cuff plethysmography system. Vasorelaxant activity was examined in vitro using isolated rat aortic rings precontracted with either Epinephrine (EP, 10 μM) or potassium chloride (KCl, 80 mM). Endothelium-dependent and endothelium-independent components were dissected by pre-incubating the rings with selective pharmacological inhibitors. Additionally, the effects of benzaldehyde on both phasic and tonic contractions induced by extracellular Ca^2+^ were assessed in EP-precontracted rings in Ca^2+^-free Krebs solution. **Key Findings**: In hypertensive animals, benzaldehyde produced a dose-related decrease in both systolic and diastolic arterial pressure. It induced concentration-dependent vasorelaxation in both endothelium-intact and -denuded aortic rings. Relaxation was also observed in KCl-precontracted rings. Vasorelaxant responses were significantly attenuated by Indomethacin, Nifedipine, and 2-Aminoethoxydiphenyl borinate (2-APB). **Significance**: These findings demonstrate that benzaldehyde lowers blood pressure through both endothelium-dependent and -independent mechanisms. These include activation of the prostacyclin (PGI_2_)/cAMP pathway, inhibition of L-type calcium channels, and blockade of store-operated calcium channels (SOCCs).

## 1. Introduction

Cardiovascular disorders (CVDs) continue to be the leading cause of mortality worldwide. In 2021 alone, they were responsible for an estimated 20.5 million deaths, with approximately 80% of these occurring in low- and middle-income countries [1]. Hypertension represents the foremost contributor to cardiovascular morbidity and premature mortality globally [2] and is conventionally defined as a systolic blood pressure (SBP) ≥140 mmHg and/or a diastolic blood pressure (DBP) ≥90 mmHg [3]. Systemic hypertension is a major risk factor for cardiovascular and renal complications, including myocardial infarction, stroke, heart failure, chronic kidney disease, atrial fibrillation, vascular dementia, and retinopathy [4]. Long-term elevation of blood pressure promotes vascular remodelling and endothelial dysfunction, thereby contributing to the development and progression of these complications [5,6]. Current antihypertensive therapy comprises several pharmacological classes that lower blood pressure through distinct mechanisms, including reduction in blood volume (thiazide and loop diuretics), inhibition of the renin–angiotensin system (ACE inhibitors), blockade of calcium influx into vascular smooth muscle (calcium-channel blockers), suppression of sympathetic activity (β-blockers, clonidine, and α-blockers), and direct vasodilatation (hydralazine and minoxidil) [7]. Despite their proven efficacy, these treatments are frequently associated with adverse effects and contraindications that limit their long-term tolerability [7].

The regulation of vascular tone is a key determinant of arterial blood pressure. Vasoconstriction of vascular smooth muscle is largely dependent on intracellular Ca^2+^ mobilisation and extracellular Ca^2+^ influx, whereas vasorelaxation may result from reduced calcium availability or activation of endothelial vasodilator pathways [8]. Among the major endothelium-derived relaxing factors, nitric oxide (NO) and prostacyclin (PGI_2_) play central roles in maintaining vascular homeostasis and regulating peripheral vascular resistance [9]. Consequently, modulation of endothelial signalling and calcium-dependent mechanisms represents an important therapeutic strategy in the management of hypertension. Several plant-derived compounds have been reported to exert antihypertensive and vasorelaxant effects through these pathways, highlighting their potential as sources of novel cardiovascular agents [10,11,12]. Compared with synthetic drugs, which often provide rapid and well-defined pharmacological effects but may be associated with adverse effects, high development costs, and limited accessibility, plant-derived compounds constitute a structurally diverse source of bioactive molecules of interest for drug discovery. Isolation, purification, and standardisation of active constituents improve the reproducibility and reliability of their pharmacological effects. Standardisation ensures consistent quality, potency, and batch-to-batch uniformity. These processes support their development and incorporation into modern drug delivery systems. Their therapeutic potential requires systematic validation through dose–response evaluation, tolerability studies, and in vitro and in vivo pharmacological investigations [13].

**Figure 1:**
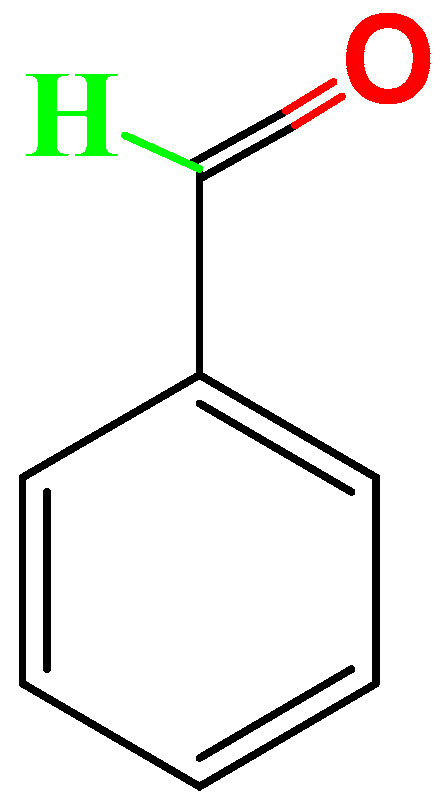
The chemical structure of benzaldehyde.

Benzaldehyde is a natural compound produced in plants by the action of specific enzymes [14,15]. Structurally, it is the simplest naturally occurring aromatic aldehyde, comprising a benzene ring bearing a single aldehyde group (Figure 1). Benzaldehyde plays an important ecological role in pollinator attraction and is responsible for the characteristic aroma of numerous plants. Its pleasant almond-like odour also contributes to the flavour of several fruits, including cherry, peach, apricot, cranberry, raspberry, and melon [14]. This molecule has been reported to display a wide range of biological activities, including insecticidal, antimicrobial, antioxidant, anti-neuroinflammatory, neuroprotective, and antitumoral effects [16,17,18]. The leaves of Prunus serotina are traditionally employed for the management of hypertension, and benzaldehyde—a major bioactive constituent of the essential oil obtained from these leaves—has been shown to exert vasorelaxant activity in vitro [19]. Such an effect suggests a potential capacity to reduce peripheral vascular resistance, an essential mechanism in blood pressure regulation. Nevertheless, to the best of our knowledge, the antihypertensive properties of benzaldehyde have not yet been investigated. The present work was therefore designed to evaluate its antihypertensive activity in normotensive and L-NAME–induced hypertensive rats and to explore the pharmacological mechanisms underlying its action on the isolated rat thoracic aorta.

## 2. Results

### 2.1. Antihypertensive Effect of Benzaldehyde

#### 2.1.1. Acute Oral Administration

Daily oral administration of Nω-nitro-L-arginine methyl ester (L-NAME) for two weeks produced a significant rise in arterial blood pressure parameters when compared with normotensive controls. Table 1 summarises the effect of benzaldehyde on blood pressure measured 6 h after a single oral dose. Neither benzaldehyde (20 or 40 mg/kg) nor furosemide modified SBP or DBP in normotensive rats. In L-NAME–induced hypertensive animals, the lower dose of benzaldehyde (20 mg/kg) produced a non-significant decrease in SBP and DBP, whereas furosemide caused a significant fall in DBP (*p <* 0.05). At the higher dose (40 mg/kg), benzaldehyde significantly reduced both SBP and DBP (*p <* 0.05) compared with the vehicle-treated control.

#### 2.1.2. Repeated Oral Administration

In the subacute experiment, normotensive and hypertensive rats received benzaldehyde at 20 or 40 mg/kg orally for seven consecutive days. The corresponding SBP and DBP values are reported in Table 2. Neither furosemide nor benzaldehyde altered blood pressure in normotensive animals. In hypertensive rats, the 20 mg/kg dose of benzaldehyde produced a significant decrease in SBP on days 2, 4, and 7 (*p <* 0.05, *p <* 0.001, and *p <* 0.0001, respectively), and an even more pronounced reduction was observed with 40 mg/kg (*p <* 0.001, *p <* 0.0001, and *p <* 0.0001, respectively). Furosemide likewise lowered SBP at all three time points (*p <* 0.0001). DBP was significantly reduced by benzaldehyde 20 mg/kg on days 4 and 7 (*p <* 0.01 and *p <* 0.001) and by benzaldehyde 40 mg/kg on days 2, 4, and 7 (*p <* 0.01, *p <* 0.001, and *p <* 0.0001). The reference drug furosemide produced comparable decreases in DBP on the same days (*p <* 0.001, *p <* 0.0001, and *p <* 0.0001) relative to the control group.

### 2.2. Vasorelaxant Activity

#### 2.2.1. Endothelium-Dependent and -Independent Relaxation

The vasorelaxant effect of benzaldehyde was assessed in isolated rat aortic rings by recording the relaxation evoked by four cumulative concentrations (0.235, 0.471, 0.707, and 0.942 mM) following precontraction with either epinephrine (EP, 10 µM) or potassium chloride (KCl, 80 mM).

**Figure 2:**
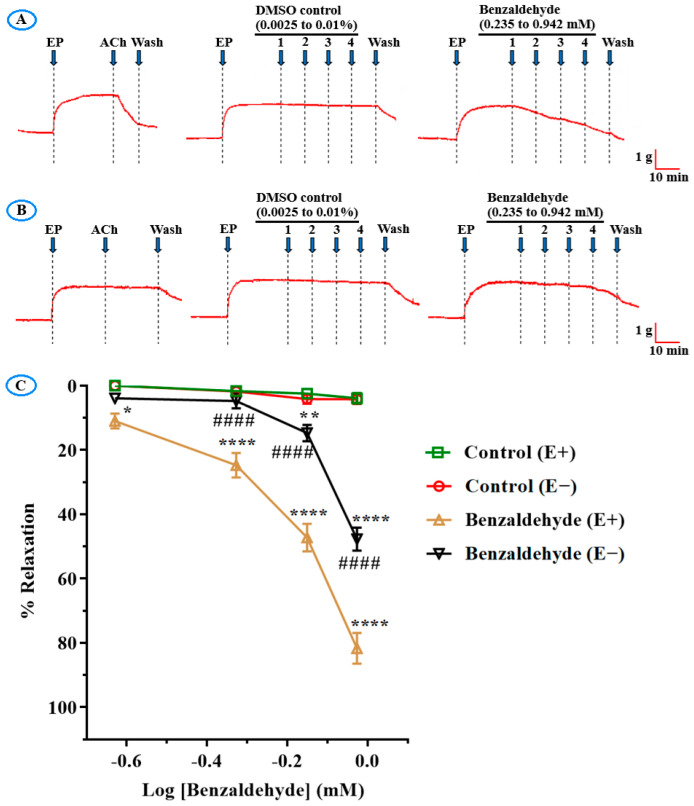
Representative tracings illustrating the concentration-dependent vasorelaxant effect of benzaldehyde and of the vehicle (DMSO) on aortic rings with intact endothelium (E+) (**A**) and without endothelium (E−) (**B**), both precontracted with epinephrine (EP, 10 µM). Numbers 1–4 refer to the cumulative concentrations applied (0.235, 0.471, 0.707, and 0.942 mM). (**C**) Concentration–response curves of benzaldehyde-induced relaxation in endothelium-intact (E+) and endothelium-denuded (E−) aortic rings precontracted with EP (10 µM). Data are mean ± SEM (*n* = 6). * *p* < 0.05; ** *p* < 0.01; **** *p* < 0.0001 vs. control. #### *p* < 0.0001 vs. endothelium-intact (E+) rings.

Once the EP-induced contraction had reached a plateau, benzaldehyde was added cumulatively to either endothelium-intact (E+) or endothelium-denuded (E−) rings. In E+ preparations, benzaldehyde produced a significant, concentration-dependent relaxation from the lowest concentration tested (0.235 mM, *p* < 0.05) and at the three subsequent concentrations (0.471, 0.707, and 0.942 mM, all *p* < 0.0001), with a maximal relaxation (Rmax) of 81.66 ± 4.71% (Figure 2). In endothelium-denuded rings, a significant relaxation was also observed, but only from 0.707 mM (*p* < 0.01) and increased further at 0.942 mM (*p* < 0.0001), reaching an Rmax of 47.69 ± 3.48%, which was significantly lower than that recorded in E+ rings (*p* < 0.0001). Endothelial removal significantly reduced the response to benzaldehyde from the second concentration onward (0.471 mM, *p* < 0.0001) (Figure 2).

**Figure 3:**
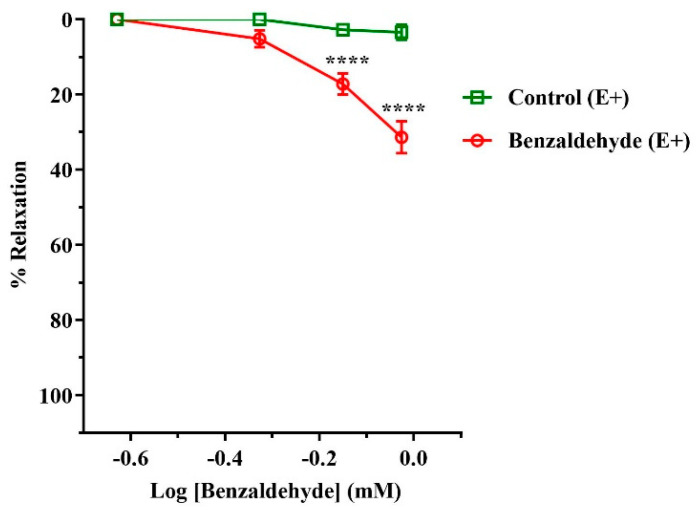
Effect of benzaldehyde on endothelium-intact (E+) aortic rings precontracted with KCl (80 mM). Data are mean ± SEM (*n* = 6). **** *p* < 0.0001 vs. control.

In endothelium-intact rings precontracted with KCl (80 mM), benzaldehyde-induced vasorelaxation became significant from the third concentration (0.707 mM, *p* < 0.0001), with an Rmax of 31.33 ± 4.13% (Figure 3).

#### 2.2.2. Effect of Pharmacological Inhibitors on Benzaldehyde-Induced Relaxation

**Figure 4:**
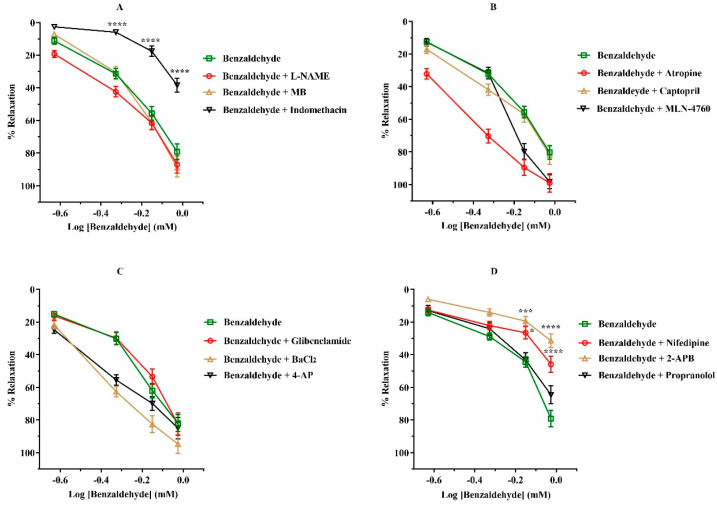
Effect of various pre-incubated drugs on benzaldehyde-induced relaxation in endothelium-intact aortic rings precontracted with EP (10 µM). Rings were relaxed by cumulative concentrations of benzaldehyde (0.235, 0.471, 0.707, and 0.942 mM) in the absence or presence of pre-incubation with: L-NAME, methylene blue, indomethacin (**A**); atropine, captopril, MLN-4760 (**B**); glibenclamide, BaCl2, 4-AP (**C**); nifedipine, 2-aminoethoxydiphenyl borate (2-APB), propranolol (**D**). Data are mean ± SEM (*n* = 6). * *p* < 0.05; *** *p* < 0.001; **** *p* < 0.0001 vs. control.

Endothelium-intact aortic rings were pre-incubated for 20 min before EP (10 µM) precontraction with one of the following agents: L-NAME (10^−4^ M), methylene blue (10^−5^ M), indomethacin (10^−5^ M), atropine (10^−5^ M), captopril (10^−5^ M), MLN-4760 (6 nM), glibenclamide (10^−5^ M), BaCl_2_ (10^−4^ M), 4-AP (10^−4^ M), nifedipine (10^−5^ M), 2-APB (10^−4^ M), or propranolol (10^−5^ M) (Figure 4). Most of the tested inhibitors did not significantly modify the relaxation evoked by benzaldehyde, with the exception of indomethacin, nifedipine, and 2-APB, which markedly attenuated the vasorelaxant response. Indomethacin reduced the relaxation from the second concentration of benzaldehyde onward (*p* < 0.0001), giving an Rmax of 38.36 ± 4.18% versus 79.08 ± 4.76% in the corresponding control (Figure 4A). Likewise, 2-APB significantly inhibited the response from the third concentration (*p* < 0.001) up to the highest one tested (*p* < 0.0001), with an Rmax of 31.42 ± 4.13% (control Rmax = 79.10 ± 5.01%) (Figure 4D). Nifedipine produced a comparable inhibition, beginning at the third concentration (*p* < 0.05) and reaching its maximal effect at the highest one (*p <* 0.0001), with an Rmax of 45.80 ± 4.79% (Figure 4D).

#### 2.2.3. Effect of Benzaldehyde on Intracellular Ca^2+^ Release and Extracellular Ca^2+^ Influx

**Figure 5:**
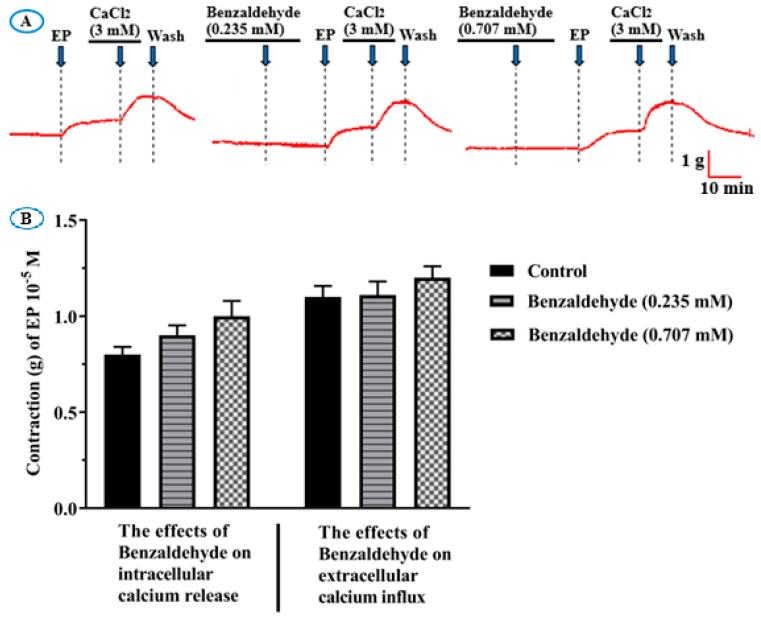
Effect of benzaldehyde on intracellular Ca^2+^ release and extracellular Ca^2+^ influx. (**A**) Representative tracings showing the effect of benzaldehyde (0.235 and 0.707 mM) on contractions induced by intracellular Ca^2+^ release and the subsequent re-addition of extracellular Ca^2+^ (3 mM) in rat aortic rings precontracted with epinephrine (10 µM) in Ca^2+^-free Krebs buffer. (**B**) Histogram showing that benzaldehyde produced no significant inhibition of either intracellular Ca^2+^ release or extracellular Ca^2+^ influx. Data are mean ± SEM (*n* = 6).

To determine whether the vasorelaxant action of benzaldehyde involves modulation of intracellular Ca^2+^ stores or extracellular Ca^2+^ entry, EP (10 µM)–induced phasic contractions and the sustained contraction triggered by re-addition of CaCl_2_ (3 mM) were recorded in EP-precontracted rings bathed in nominally Ca^2+^-free KH buffer, in the presence or absence of benzaldehyde (0.235 and 0.707 mM). Pre-incubation with benzaldehyde for 20 min did not modify the initial EP-induced phasic contraction, nor did it influence the sustained contraction evoked by extracellular CaCl_2_ (3 mM) (Figure 5).

## 3. Discussion

The aim of this study was to characterise the antihypertensive effect of benzaldehyde in normotensive and L-NAME–induced hypertensive rats over a seven-day period. Both single and repeated oral administration of benzaldehyde at 20 and 40 mg/kg produced a dose-dependent decrease in SBP and DBP in hypertensive animals, with the most marked normalisation of arterial pressure observed by day 7. In contrast, blood pressure remained unchanged in normotensive rats, an outcome that is most likely explained by the fact that the doses used fall within the range tolerated by the normal homeostatic regulation of blood pressure. At the end of the experimental period, all animals appeared healthy and showed body-weight gains comparable to those of the vehicle-treated controls. Plants represent a natural source of chemically diverse bioactive constituents with potential therapeutic value. Traditional plant-based remedies are widely used worldwide as part of primary healthcare and complementary approaches for the management of various health conditions, including hypertension [20,21]. Contraction of vascular smooth muscle (VSM) in resistance arteries and arterioles is governed by the cytoplasmic concentration of free Ca^2+^_._ A rise in cytosolic Ca^2+^—either through release from the sarcoplasmic reticulum or through influx of extracellular Ca^2+^—leads to formation of the Ca^2+^–calmodulin complex, which in turn promotes actin–myosin cross-bridge cycling and VSM contraction [22]. A reduction in vascular resistance through direct vasodilation therefore represents one of the major mechanisms by which antihypertensive drugs decrease blood pressure and maintain adequate tissue perfusion [23].

Antihypertensive agents typically achieve their vasorelaxant effect by acting on either vascular endothelial cells or vascular smooth muscle. The endothelium is a key regulator of cardiovascular homeostasis, releasing several vasodilator mediators—chiefly vasodilator prostaglandins, nitric oxide (NO), and endothelium-derived hyperpolarising factor (EDHF) [23]. In the present in vitro study, the vasorelaxant activity of this small molecule was examined in aortic rings isolated from Wistar rats in order to delineate the mechanisms underlying its antihypertensive effect. Cumulative addition of benzaldehyde (0.235–0.942 mM) produced a concentration-dependent relaxation in both endothelium-intact and endothelium-denuded thoracic aortic rings, although the magnitude of the response differed significantly between the two preparations. These observations indicate that benzaldehyde acts through both endothelium-dependent and endothelium-independent mechanisms. Moreover, the relaxation in KCl-precontracted rings was substantially weaker than that recorded in EP-precontracted rings, suggesting that voltage-dependent Ca^2+^ channels (VDCCs) play only a minor role in the vasorelaxant effect of benzaldehyde.

Endothelium-dependent vasorelaxation may arise from inhibition of ACE [24], stimulation of ACE-2 [25], activation of muscarinic receptors [26], or release of endothelium-derived relaxing factors (EDRFs), in particular those linked to prostaglandin synthesis and the NO signalling pathway [9]. Among the inhibitors targeting endothelial pathways tested in the present study (L-NAME, indomethacin, atropine, captopril, and MLN-4760), only indomethacin significantly attenuated the relaxation produced by benzaldehyde, thus confirming the involvement of an endothelium-dependent component. Endothelial cyclooxygenase-2 (COX-2) and prostacyclin synthase (PGIS) are the key enzymes involved in PGI2 biosynthesis, a pathway that is sensitive to indomethacin [27,28]. PGI2-induced relaxation is mediated through cAMP, which decreases the sensitivity of the contractile machinery to Ca^2+^ by enhancing cytoplasmic Ca^2+^ extrusion. PGI2 also produces hyperpolarization in several vascular beds by activating various subtypes of K^+^ channels [29]. The inhibitory effect of indomethacin paralleled that of mechanical removal of the endothelium, further supporting a major role for the COX/PGI2 axis.

Endothelium-independent vasorelaxation may involve activation of β-adrenoceptors [30], stimulation of soluble guanylate cyclase [31], opening of K^+^ channels [32], inhibition of extracellular Ca^2+^ influx, or suppression of Ca^2+^ release from the sarcoplasmic reticulum [33]. Among the agents acting on smooth-muscle targets (propranolol, methylene blue, nifedipine, glibenclamide, 4-AP, BaCl_2_, and 2-APB), only nifedipine and 2-APB significantly reduced the response to benzaldehyde, supporting the existence of an endothelium-independent component. Nifedipine is a selective L-type VDCC blocker [34], whereas 2-APB inhibits store-operated Ca^2+^ channels (SOCCs) and InsP3-induced Ca^2+^ release [35]. Inhibition of L-type Ca^2+^ channels [36] and SOCCs, together with attenuation of intracellular Ca^2+^ release from the endoplasmic reticulum [37], represent recurrent mechanisms by which natural products elicit vascular relaxation.

To probe the contribution of intracellular Ca^2+^ release through InsP3 receptors (IP3Rs) and of receptor-operated Ca^2+^ channel (ROCC)–mediated Ca^2+^ influx, the inhibitory effect of benzaldehyde (0.235 and 0.707 mM) was further evaluated against the EP-induced phasic contraction (10 µM) and the sustained contraction induced by addition of extracellular CaCl_2_ (3 mM) in nominally Ca^2+^-free Krebs solution [38,39]. Benzaldehyde failed to inhibit either the initial EP-evoked phasic response or the CaCl_2_-mediated sustained contraction, suggesting that its vasorelaxant effect does not involve significant blockade of IP3Rs in the sarcoplasmic reticulum or of ROCCs.

Vasodilation is fundamentally linked to a decrease in the cytoplasmic Ca^2+^ concentration in smooth-muscle cells, an event that may be triggered through several signalling pathways [12,40]. Among the major mechanisms by which natural products promote vasorelaxation are the activation of the PGI2/cAMP cascade, blockade of L-type Ca^2+^ channels, and inhibition of SOCCs [12]. Benzaldehyde is a structurally simple, low-molecular-weight natural compound, a class of molecules that occupies a central position in modern drug discovery thanks to its capacity to interact selectively with biological targets [41].

## 4. Materials and Methods

### 4.1. Experimental Animals

Healthy adult male albino rats (Wistar strain, 150–300 g) were obtained from the experimental animal facility of Missour, Morocco. The animals were maintained individually in plastic cages under standard housing conditions and fed a commercial laboratory pellet diet ad libitum. Prior to any experiment, the rats were acclimatised for at least three weeks to recover from transport-related stress.

### 4.2. Chemical Reagents and Drugs

Acetylcholine chloride, epinephrine (EP), Nω-nitro-L-arginine methyl ester (L-NAME), indomethacin, methylene blue, 2-aminoethoxydiphenyl borate (2-APB), 4-aminopyridine, barium chloride (BaCl_2_), and MLN-4760 were purchased from Sigma Chemical Co. (St. Louis, MO, USA). Atropine was supplied by ChemCruz (Santa Cruz Biotechnology, Dallas, TX, USA), while all remaining analytical-grade reagents were sourced from local distributors. Reagents and drugs were dissolved in distilled water unless otherwise indicated. Benzaldehyde (≥99%) and dimethyl sulfoxide (DMSO, ≥99.99%) were also obtained from Sigma Chemical Co.

### 4.3. Experimental Protocol

#### 4.3.1. Induction of Hypertension

Normal male albino Wistar rats (150–200 g) were used to induce hypertension by orally giving them L-NAME (60 mg/kg body weight) dissolved in 1 mL of distilled water per 100 g body weight once daily for two weeks. Distilled water was administered as a vehicle to normal control rats. The study used rats whose elevated systolic blood pressure was ≥150 mmHg, indicating that they were hypertensive [42].

#### 4.3.2. Measurement of Blood Pressure

Male Wistar rats aged 6–8 weeks and weighing 150–220 g were used in this study. Animals were randomly assigned to the experimental groups to minimise selection bias. Normotensive and hypertensive rats were divided into four groups, each consisting of six animals. The treated groups received an oral solution of benzaldehyde dissolved in 0.5% DMSO at doses of 20 and 40 mg/kg body weight. The negative control group received an equivalent volume of 0.5% DMSO, whereas the positive control group received furosemide at 20 mg/kg body weight orally. Furosemide, a loop diuretic with established antihypertensive and diuretic activity, was used as a reference drug. Blood pressure parameters were measured using a non-invasive caudal plethysmography system connected to a PowerLab data acquisition unit and LabChart 5.0 software (Harvard, Boyer, Casablanca, Morocco). Measurements were performed in all groups under identical experimental conditions after a single oral administration and then daily for 7 consecutive days during repeated administration. Before each measurement session, rats were maintained at approximately 35 °C for 15–20 min to ensure adequate tail blood flow and reliable recordings. Animals were then anaesthetised with ether and placed in restrainers equipped with a tail-cuff sensor. The cuff was inflated to a pressure exceeding 200 mmHg and gradually deflated while pulse signals were recorded through the PowerLab system and analysed using LabChart 5.0 software. Three consecutive measurements were obtained for each rat. Systolic blood pressure (SBP) and mean blood pressure (MBP) were recorded directly, whereas diastolic blood pressure (DBP) was calculated using the following equation: $DBP=\frac{3\times MBP-SBP}{2}$.

All measurements were conducted at the same time of day throughout the treatment period [43].

#### 4.3.3. Vascular Reactivity Studies and Mechanistic Assessment

Thoracic aortas were isolated from anesthetised [44] male rats (250–300 g), cleaned, and cut into 3–4 mm rings. Rings were mounted in organ baths filled with Krebs–Henseleit solution, oxygenated and maintained at 37 °C, and connected to a force transducer system (PowerLab) for isometric tension recording. After equilibration under 2 g resting tension, aortic rings were contracted using Epinephrine (EP, 10 µM) or potassium chloride (KCl, 80 mM), and cumulative concentrations of benzaldehyde (0.235, 0.471, 0.707, and 0.942 mM) were added to assess vasorelaxant effects. Endothelial integrity was evaluated using Acetylcholine (10 μM), and mechanisms of action were investigated through 20 min preincubation with pharmacological inhibitors targeting major endothelial and smooth muscle pathways, including L-NAME (10^−4^ M, nitric oxide synthase inhibitor), indomethacin (10^−5^ M, prostaglandin synthesis inhibitor), atropine (10^−5^ M, muscarinic receptor antagonist), captopril (10^−5^ M, angiotensin-converting enzyme inhibitor), MLN-4760 (6 nM, ACE2 inhibitor), glibenclamide (10^−5^ M, ATP-sensitive K^+^ channel blocker), nifedipine (10^−5^ M, L-type Ca^2+^ channel blocker), methylene blue (10^−5^ M, guanylate cyclase inhibitor), propranolol (10^−5^ M, β-adrenergic receptor blocker), 2-aminoethoxydiphenyl borinate (2-APB, 10^−4^ M, inhibitor of store-operated Ca^2+^ entry and IP_3_-mediated Ca^2+^ release), barium chloride (BaCl_2_, 10^−5^ M, inward rectifier K^+^ channel blocker), and 4-aminopyridine (4-AP, 10^−4^ M, voltage-dependent K^+^ channel blocker). Benzaldehyde was solubilised in 1% DMSO, with the vehicle added at a final cumulative concentration not exceeding 0.025%. The DMSO concentrations were selected based on previous studies, ensuring that they did not exert any relaxation effect on aortic rings [38,45]. Control experiments confirmed the absence of a significant effect of the solvent on the vascular tone of the aortic rings. The relaxant response was expressed as a percentage of the contraction induced by EP or KCl. This protocol was conducted as previously described [43]. The vasorelaxant response was expressed as the percentage of contraction induced by EP or KCl, calculated as:

$$Relaxation\;(\%)=\frac{tension\;induced\;by\;vasodilator}{tension\;induced\;by\;EP}\times 100$$

### 4.4. Statistical Analysis

Data are presented as mean ± SEM. Comparisons were performed using one-way and two-way ANOVA followed by Bonferroni’s post hoc multiple comparison test (GraphPad Prism version 8.0, GraphPad Software Inc., San Diego, CA, USA). A *p*-value < 0.05 was considered statistically significant.

## 5. Conclusions

Benzaldehyde, the simplest naturally occurring aromatic aldehyde, is a plant-derived compound that contributes to the characteristic aroma of numerous plants and fruits and has been credited with multiple pharmacological activities. The present investigation demonstrates that benzaldehyde elicits a dose-dependent antihypertensive effect in L-NAME–induced hypertensive rats. This action appears to involve both endothelium-dependent vasorelaxation, mediated by activation of the prostacyclin (PGI2)/cAMP pathway, and endothelium-independent vasorelaxation, achieved through blockade of L-type voltage-dependent Ca^2+^ channels and inhibition of store-operated Ca^2+^ channels (SOCCs). Taken together, these findings suggest that benzaldehyde may be considered a promising candidate for further development as a functional ingredient or therapeutic agent for the prevention and management of hypertension.

## Figures and Tables

**Table 1:** Effect of a single oral administration of benzaldehyde (20 and 40 mg/kg) on systolic and diastolic arterial blood pressure (mm Hg) in normotensive and L-NAME–induced hypertensive rats. Values are expressed as mean ± SEM (*n* = 6). * *p <* 0.05; t: time in hours.

		**Systolic Blood Pressure (mm Hg)**			
		**Control**	**Benzaldehyde (20 mg/kg)**	**Benzaldehyde (40 mg/kg)**	**Furosemide (20 mg/kg)**
Normal	t0	130.00 ± 7.00	128.00 ± 5.00	130.00 ± 6.00	122.00 ± 7.00
	t6	128.00 ± 6.00	126.00 ± 6.00	122.00 ± 7.00	112.00 ± 7.00
L-NAME	t0	182.00 ± 8.00	180.00 ± 10.00	181.00 ± 8.00	171.00 ± 6.00
	t6	178.00 ± 7.00	158.00 ± 8.00	149.00 ± 9.00 *	143.00 ± 7.00
		**Diastolic Blood Pressure (mm Hg)**			
		**Control**	**Benzaldehyde (20 mg/kg)**	**Benzaldehyde (40 mg/kg)**	**Furosemide (20 mg/kg)**
Normal	t0	108.00 ± 8.00	104.00 ± 4.00	106.00 ± 7.00	107.00 ± 6.00
	t6	103.00 ± 7.00	104.00 ± 7.00	102.00 ± 6.00	97.00 ± 6.00
L-NAME	t0	148.00 ± 8.00	148.00 ± 6.00	150.00 ± 8.00	143.00 ± 5.00
	t6	144.00 ± 6.00	131.00 ± 6.00	122.00 ± 7.00 *	118.00 ± 4.00 *

**Table 2:** Effect of repeated oral administration of benzaldehyde (20 and 40 mg/kg) on systolic and diastolic arterial blood pressure (mm Hg) in normotensive and L-NAME–induced hypertensive rats. Values are expressed as mean ± SEM (*n* = 6). * *p <* 0.05; ** *p <* 0.01; *** *p <* 0.001; **** *p <* 0.0001; D: day.

		**Systolic Blood Pressure (mm Hg)**			
		**Control**	**Benzaldehyde (20 mg/kg)**	**Benzaldehyde (40 mg/kg)**	**Furosemide (20 mg/kg)**
Normal	D0	130.00 ± 7.00	128.00 ± 5.00	130.00 ± 6.00	122.00 ± 7.00
	D2	129.00 ± 6.00	124.00 ± 6.00	120.00 ± 5.00	118.00 ± 5.00
	D4	127.00 ± 8.00	123.00 ± 7.00	118.00 ± 6.00	110.00 ± 6.00
	D7	128.00 ± 6.00	121.00 ± 8.00	116.00 ± 8.00	102.00 ± 7.00
L-NAME	D0	182.00 ± 7.00	180.00 ± 10.00	181.00 ± 8.00	171.00 ± 6.00
	D2	179.00 ± 8.00	152.00 ± 7.00 *	141.00 ± 7.00 ***	125.00 ± 5.00 ****
	D4	176.00 ± 7.00	144.00 ± 5.00 ***	132.00 ± 6.00 ****	121.00 ± 6.00 ****
	D7	175.00 ± 6.00	135.00 ± 8.00 ****	128.00 ± 7.00 ****	110.00 ± 7.00 ****
		**Diastolic Blood Pressure (mm Hg)**			
		**Control**	**Benzaldehyde (20 mg/kg)**	**Benzaldehyde (40 mg/kg)**	**Furosemide (20 mg/kg)**
Normal	D0	108.00 ± 8.00	104.00 ± 4.00	106.00 ± 7.00	107.00 ± 6.00
	D2	104.00 ± 7.00	102.00 ± 7.00	100.00 ± 7.00	103.00 ± 8.00
	D4	102.00 ± 6.00	100.00 ± 5.00	98.00 ± 5.00	100.00 ± 5.00
	D7	106.00 ± 8.00	97.00 ± 6.00	95.00 ± 8.00	93.00 ± 7.00
L-NAME	D0	145.00 ± 8.00	148.00 ± 6.00	150.00 ± 8.00	143.00 ± 5.00
	D2	140.00 ± 7.00	126.00 ± 6.00	119.00 ± 6.00 **	106.00 ± 5.00 ***
	D4	138.00 ± 7.00	116.00 ± 8.00 **	113.00 ± 5.00 ***	99.00 ± 6.00 ****
	D7	136.00 ± 8.00	110.00 ± 6.00 ***	101.00 ± 7.00 ****	92.00 ± 7.00 ****

## Data Availability

The original contributions presented in this study are included in the article. Further inquiries can be directed to the corresponding authors.
